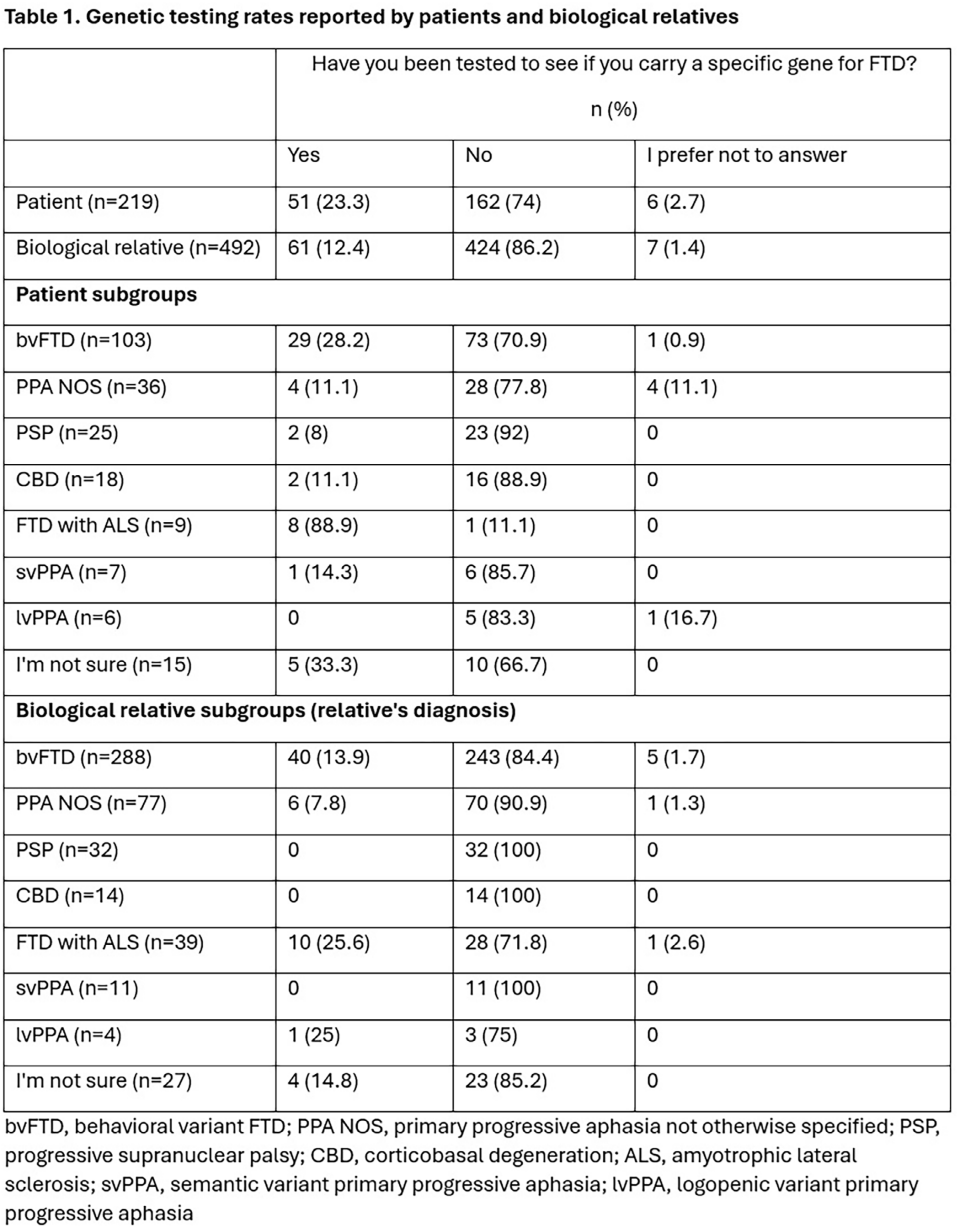# Genetic testing rates of individuals diagnosed with FTD and close biological relatives assessed in the FTD Insights Survey

**DOI:** 10.1002/alz70858_107543

**Published:** 2025-12-25

**Authors:** Devon M Chenette, Stella McCaughey, Robert Reinecker, Carrie F Milliard, Tiffany W Chow, Penny A Dacks

**Affiliations:** ^1^ Alector, South San Francisco, CA, USA; ^2^ FTD Disorders Registry, King of Prussia, PA, USA; ^3^ The Association for Frontotemporal Degeneration, King of Prussia, PA, USA

## Abstract

**Background:**

Frontotemporal dementia (FTD) is a heterogenous disorder characterized by early age of onset and changes in behavior or language. It is estimated that approximately 20% of patients have an autosomal dominant presentation. Mutations in the *GRN*, *MAPT*, or *C9orf72* genes account for the majority of genetic FTD.

**Method:**

The FTD Insights Survey was developed and executed by the Association for Frontotemporal Degeneration (AFTD) and the FTD Disorders Registry. This dataset contains 1,800 responses (US, UK, Canada) and is available to researchers. Responses to “Have you been tested to see if you carry a specific gene for FTD” were analyzed. Data was assessed for 1) FTD patients who selected “I am diagnosed with FTD” and 2) relatives who selected “I have a close biological relative with FTD” and didn’t select “I am diagnosed with FTD”.

**Result:**

Twenty‐three percent (23.3%, *n* = 51) of 219 patients received genetic testing. Individuals diagnosed with FTD with ALS (*n* = 9) reported the highest rates of genetic testing (88.9%, *n* = 8). A greater percentage of patients who completed genetic testing reported having at least one biological relative diagnosed with FTD compared to those who did not receive genetic testing (44.2% vs.12% respectively).

Twelve percent (12.4%, *n* = 61) of 492 biological relatives received genetic testing. Relatives of individuals diagnosed with FTD with ALS (*n* = 39) reported the highest rates of genetic testing (25.6%, *n* = 10). Of relatives who responded yes (*n* = 95) to the question, “Does your family carry a gene for FTD”, 49.5% (*n* = 47) received genetic testing.

**Conclusion:**

Less than a quarter of FTD patients received genetic testing despite genetic forms comprising a notable percentage of all FTD cases. Highest rates were reported for individuals diagnosed with FTD with ALS, and rates were higher with known family history. Low rates of genetic testing may exacerbate challenges facing families impacted by FTD such as assessing familial risk of developing FTD and determining eligibility for investigational drugs in development for genetic forms of FTD. Additional research is needed to better understand the accessibility of genetic testing and counseling. Taken together, these findings highlight the need to further raise awareness of genetic FTD with healthcare providers, patients, and biological relatives.